# Enhanced firing of locus coeruleus neurons and SK channel dysfunction are conserved in distinct models of prodromal Parkinson’s disease

**DOI:** 10.1038/s41598-022-06832-1

**Published:** 2022-02-24

**Authors:** Lina A. Matschke, Marlene A. Komadowski, Annette Stöhr, Bolam Lee, Martin T. Henrich, Markus Griesbach, Susanne Rinné, Fanni F. Geibl, Wei-Hua Chiu, James B. Koprich, Jonathan M. Brotchie, Aytug K. Kiper, Amalia M. Dolga, Wolfgang H. Oertel, Niels Decher

**Affiliations:** 1 Institute for Physiology and Pathophysiology, Vegetative Physiology and Marburg Center for Mind, Brain and Behavior - MCMBB, Philipps-University Marburg, 35037 Marburg, Germany; 2 Clinic for Neurology, Philipps-University Marburg, 35043 Marburg, Germany; 3 Krembil Research Institute, Toronto Western Hospital, University Health Network, 8KD402, Toronto, ON M5T 2S8 Canada; 4 Faculty of Science and Engineering, Groningen Research Institute of Pharmacy, Department of Molecular Pharmacology, University of Groningen, 9713 AV Groningen, The Netherlands; 5 Hertie Senior Research Professor of the Charitable Hertie Foundation, 60323 Frankfurt am Main, Germany

**Keywords:** Biophysics, Physiology, Ion channels in the nervous system, Molecular neuroscience, Parkinson's disease

## Abstract

Parkinson’s disease (PD) is clinically defined by the presence of the cardinal motor symptoms, which are associated with a loss of dopaminergic nigrostriatal neurons in the substantia nigra pars compacta (SNpc). While SNpc neurons serve as the prototypical cell-type to study cellular vulnerability in PD, there is an unmet need to extent our efforts to other neurons at risk. The noradrenergic locus coeruleus (LC) represents one of the first brain structures affected in Parkinson’s disease (PD) and plays not only a crucial role for the evolving non-motor symptomatology, but it is also believed to contribute to disease progression by efferent noradrenergic deficiency. Therefore, we sought to characterize the electrophysiological properties of LC neurons in two distinct PD models: (1) in an in vivo mouse model of focal α-synuclein overexpression; and (2) in an in vitro rotenone-induced PD model. Despite the fundamental differences of these two PD models, α-synuclein overexpression as well as rotenone exposure led to an accelerated autonomous pacemaker frequency of LC neurons, accompanied by severe alterations of the afterhyperpolarization amplitude. On the mechanistic side, we suggest that Ca^2+^-activated K^+^ (SK) channels are mediators of the increased LC neuronal excitability, as pharmacological activation of these channels is sufficient to prevent increased LC pacemaking and subsequent neuronal loss in the LC following in vitro rotenone exposure. These findings suggest a role of SK channels in PD by linking α-synuclein- and rotenone-induced changes in LC firing rate to SK channel dysfunction.

## Introduction

Parkinson’s disease (PD), the second most common neurodegenerative disorder, is a progressive, age-related movement disorder. It is characterized by the accumulation of α-synuclein (αSyn) positive eosinophilic neuronal inclusions, termed Lewy pathology, and neurodegeneration. Importantly, even in late stage PD patients, Lewy pathology and neuronal cell loss are confined to certain vulnerable brain regions^1,2^. Affected neurons share a set of morphological and functional traits going along with a high energetic and metabolic demand. These traits include e.g. long and highly branched axons, a large number of neurotransmitter release sites, and slow tonic pacemaking activity^2^. While dopaminergic substantia nigra (SN) neurons serve as the prototypical cell-type to study cellular vulnerability in PD, there is an unmet need to extend our efforts to study other neurons at risk. The noradrenergic locus coeruleus (LC), a small nucleus in the pontine brainstem which constitutes the major source of noradrenaline (NA) in the central nervous system, has been identified as a key structure in the prodromal or premotor stage of PD^3,4^. Results from previous studies show that LC cells suffer from αSyn aggregation and Lewy pathology formation several years if not decades before the degeneration of dopaminergic SN neurons^5,6^. Further, LC cell loss might even exceed nigral neurodegeneration^7^. LC dysfunction and neurodegeneration are associated with several non-motor symptoms of PD, such as anxiety, depression or reduced arousal^8,9^. Additionally, noradrenaline released from LC efferent neurons has been shown to exert potent anti-inflammatory effects^10,11^. Given the central role of neuroinflammation in neurodegenerative disorders^12^, it is not surprising that LC ablation has been shown to aggravate nigral neurodegeneration in toxin models of PD^13–15^, whereas genetic or pharmacological enhancement of noradrenergic neurotransmission was able to mitigate midbrain pathology and PD symptoms^11,16,17^. Hence, affection of the LC-noradrenergic system during PD has been proposed to not only contribute to evolving non-motor symptomatology, but due to efferent noradrenergic deficiency and the absent anti-inflammatory effect also to disease progression. Thus, studies aimed at investigating LC neuronal (dys)function prior to marked neurodegeneration may not only provide important insights into the mechanisms leading to noradrenergic imbalance and LC cell loss, but foster identification of potential cellular targets for disease modifying therapy approaches.

Therefore, we sought to characterize the electrophysiological alterations of LC neurons at the single cell level in two distinct PD models. To this end, we conducted whole-cell patch clamp experiments of LC neurons in: (1) a well characterized in vivo mouse model relying on viral vector mediated focal overexpression of human wild-type (WT) or human A53T-mutated αSyn^18^; and (2) in an in vitro PD model based on the application of the mitochondrial complex I inhibitor rotenone. Our results yielded three key observations: First, despite the fundamental differences between these two models, αSyn overexpression as well as rotenone exposure led to a significantly accelerated autonomous LC pacemaker frequency which was associated with a marked decrease of the afterhyperpolarization amplitude. Second, we identified small-conductance Ca^2+^-activated K^+^ (SK) channels as mediators of the significantly increased excitability of LC neurons in both models. Third, our experiments suggest that SK channels might represent valuable pharmacological targets for disease modifying therapy approaches, since application of the SK channel activator, 6,7-dichloro-1H-indole-2,3-dione 3-oxime (NS309) not only normalized LC firing frequency, but even counteracted neuronal LC loss in the in vitro rotenone model. These novel findings raise the possibility that SK channels are possible mediators of LC neurodegeneration in PD.

## Results

### Progressive acceleration of the pacemaker frequency after overexpression of human mutated αSyn^A53T^ in LC neurons

**Figure 1:**
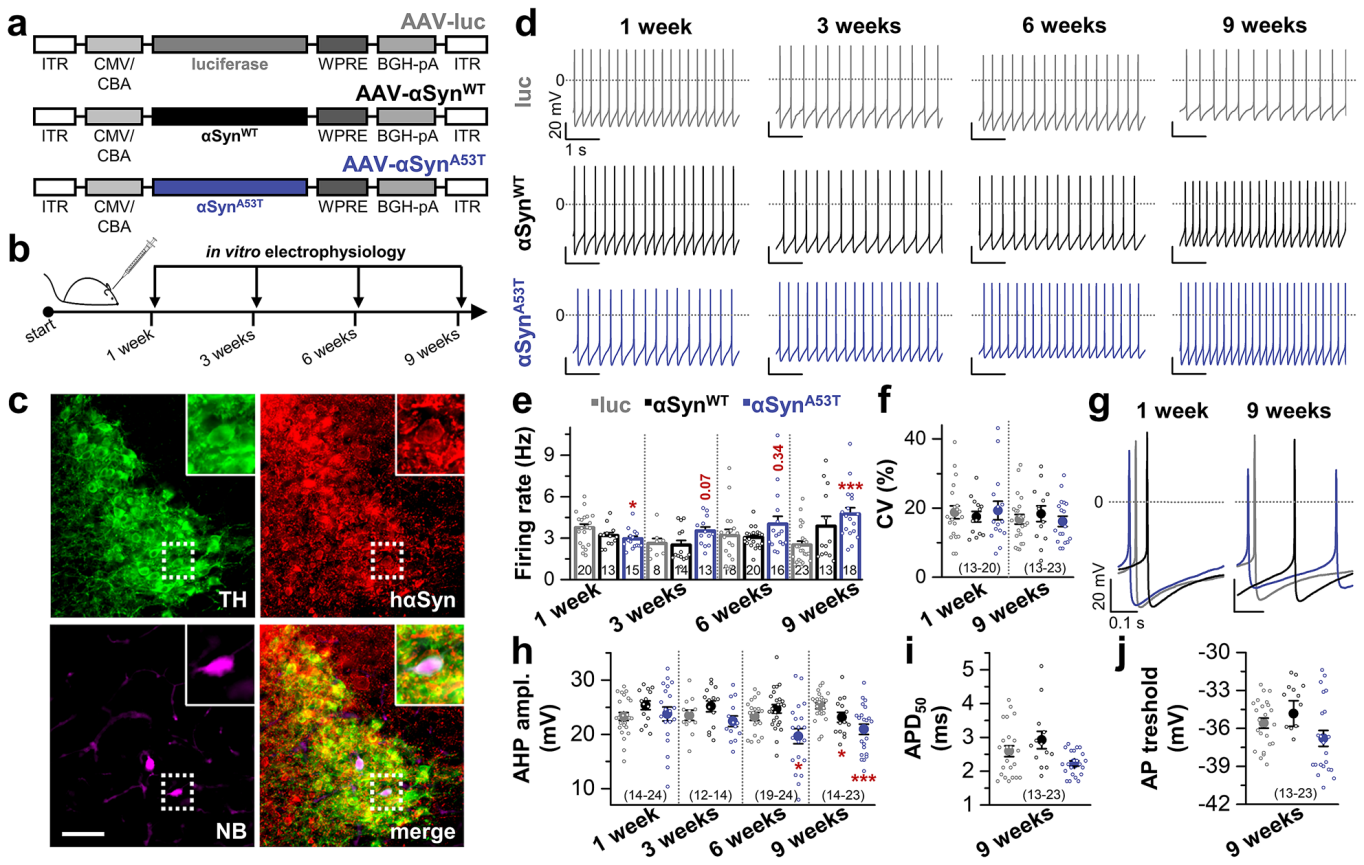
rAAV mediated αSyn^A53T^ overexpression induces acceleration of the intrinsic pacemaker frequency in LC neurons. (**a**) rAAV1/2 vectors containing a chicken β-actin promoter hybridized with a CMV immediate early enhancer sequence (CMV/CBA) were injected into the right LC to drive expression of αSyn^WT^, αSyn^A53T^, or luc (control). WPRE, woodchuck hepatitis virus posttranscriptional regulatory element; BGH-pA, bovine growth hormone polyadenylation sequence; ITR, inverted terminal repeat. (**b**) Scheme illustrating the experimental design of the study. Mice were consecutively sacrificed 1, 3, 6 and 9 weeks after injection of rAAV vectors for in vitro electrophysiology. (**c**) Representative triple immunofluorescence staining for TH (green), human αSyn (red), or neurobiotin (NB, magenta). Co-localization identifies patched, NB-filled neurons as TH- and αSyn-positive. Scale bar: 100 µm. (**d**) Example recordings of spontaneously active luc, αSyn^WT^ or αSyn^A53T^ overexpressing LC neurons in the whole-cell current clamp configuration at 1, 3, 6 and 9 weeks after injection in the presence of synaptic blockers. (**e**) Quantification of the pacemaker frequency revealed a significant acceleration of action potential firing 9 weeks post-injection in αSyn^A53T^ overexpressing animals compared to luc controls*.* (**f**) Analysis of the CV as a measure for regularity of the data shown in (**e**). (**g**) Representative AP of luc (grey), αSyn^WT^ (black) and αSyn^A53T^ (blue) overexpressing animals 1 and 9 weeks post-injection. The AP waveform of αSyn^A53T^ overexpressing neurons showed time-dependent alterations. (**h**–**j**) Analyses of basic AP properties revealed a time-dependent reduction of the AHP amplitude in αSyn^WT^ overexpressing mice at 9 weeks post-injection and in αSyn^A53T^ overexpressing mice at 6 and 9 weeks post-injection (**h**), whereas APD_50_ (**i**) and AP threshold (**j**) remained unchanged. Data are represented as mean ± SEM and also as individual data points. *p < 0.05; ***p < 0.001. [(**e**) 1 week: unpaired Student’s *t*-test vs. luc; 3 weeks: αSyn^WT^, Mann–Whitney-U test vs. luc/αSyn^A53T^, unpaired Student’s *t*-test vs. luc; 6 weeks: Mann–Whitney-U test vs. luc; 9 weeks: αSyn^WT^, Mood’s median test vs. luc/αSyn^A53T^, Mann–Whitney-U test vs. luc. (**f**) unpaired Student’s *t*-test vs. corresponding luc. (**h**) 1 week and 3 weeks: unpaired Student’s *t*-test vs. corresponding luc; 6 and 9 weeks: αSyn^WT^, unpaired Student’s *t*-test vs. corresponding luc/αSyn^A53T^, Welch’s *t*-test vs. corresponding luc. (**i**,**j**) Mann–Whitney-U test vs. corresponding luc].

To study whether and in which time-frame overexpression of αSyn alters the firing behavior of LC neurons, recombinant adeno-associated viral (rAAV) vectors containing the gene for either human wild-type αSyn (AAV-αSyn^WT^), human mutant αSyn (AAV-αSyn^A53T^) or luciferase as a control (AAV-luc) (Fig. 1a) were injected into the right LC of C57BL/6N wild-type mice. In a recent study, we already confirmed that these vectors lead to equal transduction rates of LC neurons^18^. Mice were consecutively sacrificed 1, 3, 6 and 9 weeks post-injection and electrophysiological recordings of LC neurons were performed in acute brainstem slices (Fig. 1b). To verify the noradrenergic nature of the investigated neurons and probe that they express the injected proteins, neurons were filled with NB via the pipette during patch clamp recording and post hoc co-stained for TH, the rate limiting enzyme in noradrenaline synthesis, and human αSyn (hαSyn) (Fig. 1c).

In a first set of experiments, spike trains in the whole-cell current clamp configuration were recorded to elucidate alterations of the spontaneous pacemaker activity of LC neurons. Representative recordings of spontaneously active luc, αSyn^WT^ and αSyn^A53T^ overexpressing LC neurons are displayed in Fig. 1d for each time-point. At one week post-injection, each group showed spontaneous pacemaker activity with frequencies of ~ 3 Hz, which corresponds to the pacemaker frequency described for non-manipulated LC neurons^19–21^. However, over the time-frame of 9 weeks, a gradual acceleration of firing frequency was detected in αSyn^A53T^, but not in luc overexpressing LC neurons. Note that for αSyn^WT^ overexpressing neurons there was also a tendency towards increased firing rates after 9 weeks compared to luc injected animals which did however not reach statistical significance (Fig. 1e and Supplementary Fig. S1). Notably, after 9 weeks, the mean pacemaker frequency of αSyn^A53T^ overexpressing neurons was approximately two-fold higher than that of luc control (Fig. 1e and Supplementary Fig. S1). In order to analyze the precision of pacemaking, the coefficient of variation (CV) was measured as previously described^22,23^. Under control conditions, the CV of LC neurons was ~ 18%, reflecting high precision of the pacemaker mechanism. αSyn^WT^ or αSyn^A53T^ did not alter the precision of pacemaking in LC neurons, since the CV was not significantly changed (Fig. 1f). However, we observed clear time-dependent alterations of the action potential (AP) waveform of αSyn^A53T^ and αSyn^WT^ overexpressing neurons (Fig. 1g). Compared to luc controls, the afterhyperpolarization (AHP) amplitude was significantly reduced in αSyn^A53T^ overexpressing LC neurons at 6 and 9 weeks post-injection, and in αSyn^WT^ overexpressing mice at 9 weeks post-injection (Fig. 1h). In contrast, AP duration at 50% of the peak (APD_50_) and AP threshold remained unchanged (Fig. 1i,j). In summary, our data show that overexpression of αSyn^A53T^ led to a time-dependent amplification of the LC neurons’ excitability.

### αSyn induced, progressive alterations of Ca^2+^- and small conductance Ca^2+^-activated K^+^ currents in LC neurons

**Figure 2:**
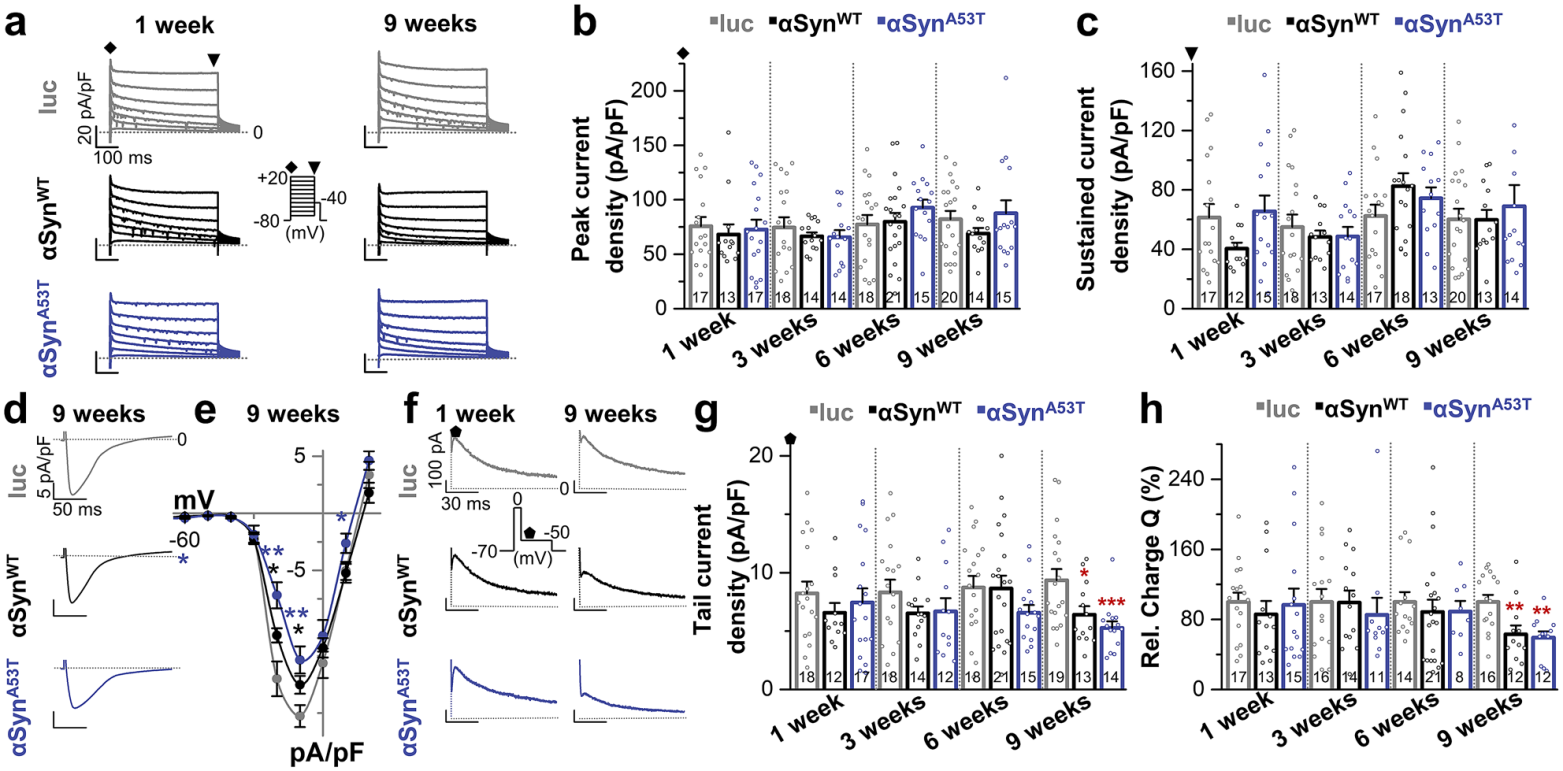
rAAV mediated αSyn^A53T^ overexpression induces alterations of Ca^2+^- and small conductance Ca^2+^-activated K^+^ currents in LC neurons. (**a**) Averaged whole-cell voltage clamp recordings of K^+^ currents 1 and 9 weeks post-injection activated by a voltage protocol with 500 ms steps ranging from − 70 to + 20 mV starting from a holding potential of – 80 mV. (**b**) Peak current densities of *I*_A_ were ascertained from the start of each voltage step (indicated by rhombus in **a**) and quantified for each time-point after injection of viral vectors. (**c**) Peak current densities of *I*_sus_ were ascertained from the end of each voltage step (indicated by triangle in **a**). For *I*_sus_ current recordings the membrane potential was depolarized to − 40 mV for 100 ms prior to voltage steps to remove *I*_A_ current components. (**d**) Average of Ca^2+^ current recordings 9 weeks after injection of viral vectors derived from a voltage step to − 10 mV. (**e**) I/V relationships of peak Ca^2+^ currents 9 weeks after injection, using the same voltage protocol as in (**a**). Ca^2+^ currents were significantly reduced 9 weeks post-injection in both αSyn^WT^ and αSyn^A53T^ injected mice (luc: n = 14, αSyn^WT^: n = 28, αSyn^A53T^: n = 12). (**f**) Representative *I*_AHP_ currents, activated by a two-pulse voltage clamp protocol as described previously^22^. The representative *I*_AHP_ currents 9 weeks after injection already display a drastic reduction in αSyn overexpressing neurons compared to luc. (**g**,**h**) Quantification of *I*_AHP_ current densities revealed significant reduction of AHP outward currents (**g**) and relative charge Q (**h**) for αSyn^WT^ and αSyn^A53T^ 9 weeks after injection. Data are presented as mean ± SEM and also as individual data points. *p < 0.05; **p < 0.01; ***p < 0.001. [(**b**) 1 week: αSyn^WT^, Mann–Whitney-U test vs. luc/αSyn^A53T^, unpaired Student’s *t*-test vs. luc; 3 weeks: Welch’s *t*-test vs. luc; 6 weeks: unpaired Student’s *t*-test vs. luc; 9 weeks: Mann–Whitney-U test vs. luc. (**c**) 1 week and 6 weeks: Mann–Whitney-U test vs. corresponding luc; 3 weeks: αSyn^WT^, Welch’s *t*-test vs. luc/αSyn^A53T^, Mann–Whitney-U test vs. luc; 9 weeks: αSyn^WT^, unpaired Student’s *t*-test vs. luc/αSyn^A53T^, Mann–Whitney-U test vs. luc. (**e**) Mann–Whitney-U test vs. luc. (**g**) 1 week: αSyn^WT^, Mann–Whitney-U test vs. luc/αSyn^A53T^, unpaired Student’s *t*-test vs. luc; 3 weeks: αSyn^WT^, Welch’s *t*-test vs. luc/αSyn^A53T^, unpaired Student’s *t*-test vs. luc; 6 weeks: αSyn^WT^, unpaired Student’s *t*-test vs. luc/αSyn^A53T^, Welch’s *t*-test vs. luc; 9 weeks: αSyn^WT^, unpaired Student’s *t*-test vs. luc/αSyn^A53T^, Mann–Whitney-U test vs. luc. (**h**) 1 week and 6 weeks: Mann–Whitney-U test vs. corresponding luc; 3 weeks: αSyn^WT^, unpaired Student’s *t*-test vs. luc/αSyn^A53T^, Mann–Whitney-U test vs. luc; 9 weeks: unpaired Student’s *t*-test vs. luc].

To investigate the underlying biophysical mechanisms of αSyn-induced increase of LC neuron firing, we analyzed whether distinct pacemaker currents were altered in these models. We have previously described that L- and T-type voltage-dependent Ca^2+^ (Cav) channels, as well as SK channels play important roles in the regulation of LC pacemaker frequency and shape of the AHP^21,22^. In addition, A-type and delayed rectifier K^+^ channels are known to be functionally expressed in LC neurons^24,25^. Therefore, we performed whole-cell voltage clamp recordings utilizing different voltage protocols to analyze K^+^, Ca^2+^, and Ca^2+^ activated K^+^ currents at each time-point following overexpression of luc, αSyn^WT^ and αSyn^A53T^. In a first step, potential changes of rapidly inactivating K^+^ currents (*I*_A_) were investigated (Fig. 2a). Quantification of *I*_A_ peak current densities revealed no significant changes in either αSyn^WT^ or in αSyn^A53T^ overexpressing LC neurons compared to luc controls at any investigated time-point (Fig. 2b). Closer consideration of the current–voltage (I/V) relationships of *I*_A_ currents after 1, 3, 6 and 9 weeks post-injection supported the finding that these currents remained almost unchanged (Supplementary Fig. S2). Next, we analyzed peak current densities of delayed-rectifier/sustained K^+^ currents (*I*_sus_). However, peak current densities (Fig. 2c) as well as I/V relationships (Supplementary Fig. S2) remained unchanged. These results indicate that voltage-dependent K^+^ channels of LC neurons are not influenced by αSyn overexpression and most likely do not contribute to the observed increase of excitability.

Next, we investigated whether Ca^2+^ inward currents may be altered due to αSyn overexpression. As depicted in the average currents (Fig. 2d) and the I/V curves (Fig. 2e), at 9 weeks post-injection LC neurons of αSyn^WT^ and αSyn^A53T^ overexpressing mice exhibited Ca^2+^ currents which were significantly reduced in a voltage range between − 20 mV and + 10 mV compared to luc controls (Fig. 2e). Subsequently, we investigated whether Ca^2+^ activated K^+^ currents are altered due to αSyn overexpression and isolated currents flowing during the AHP of LC neurons (*I*_AHP_), as it has already been shown that AHP currents are mainly mediated by SK channels^22^. The representative AHP currents in Fig. 2f display a drastic reduction of AHP current amplitudes after 9 weeks of overexpression in both αSyn groups compared to luc overexpressing neurons. Quantification of the *I*_AHP_ peak current densities confirmed a significant reduction after 9 weeks of both αSyn groups compared to luc overexpressing neurons (Fig. 2g and Supplementary Fig. S3). Finally, as shown in Fig. 2h, we analyzed the percentage of the charge (Q), assessed from the integral of the AHP current recordings (*I*_AHP_), at all time-points. The relative charge transfer through the AHP mediating channels was after 9 weeks of overexpression significantly decreased in both αSyn^WT^ and αSyn^A53T^ overexpressing LC neurons. Consistent with the more drastic increase of firing rates in αSyn^A53T^ injected mice, the effects on Ca^2+^ and AHP currents were also more pronounced in this group. Based on our previous observation that SK channels regulate LC neuron pacemaking by shaping of the AHP^22^, it is reasonable to assume that an overtime augmenting dysfunction of these channels, in combination with altered Ca^2+^ inward currents, may be causative for the increased excitability of αSyn^WT^ and αSyn^A53T^ overexpressing LC neurons.

### Electrophysiological alterations of LC neurons in an in vitro model of rotenone toxicity resemble those of αSyn overexpression

In a next set of experiments, we aimed to investigate if these electrophysiological alterations are specific for the αSyn overexpression model, or whether they might be conserved in other PD models. To do so, we used an in vitro model with the neurotoxin rotenone. Rotenone, a commonly used pesticide^26^, inhibits complex I of the electron transport chain of the mitochondria, causing dopaminergic and noradrenergic neurodegeneration alongside with parkinsonian symptoms^27–29^. However, it is still unclear if and how rotenone influences the electrophysiological properties of LC neurons. We addressed this question using two different approaches: (1) by adding 1 µM rotenone to the bath solution to assess direct effects, and (2) by incubating brainstem slices in 1 µM rotenone for 120 min prior to electrophysiological recording.

**Figure 3:**
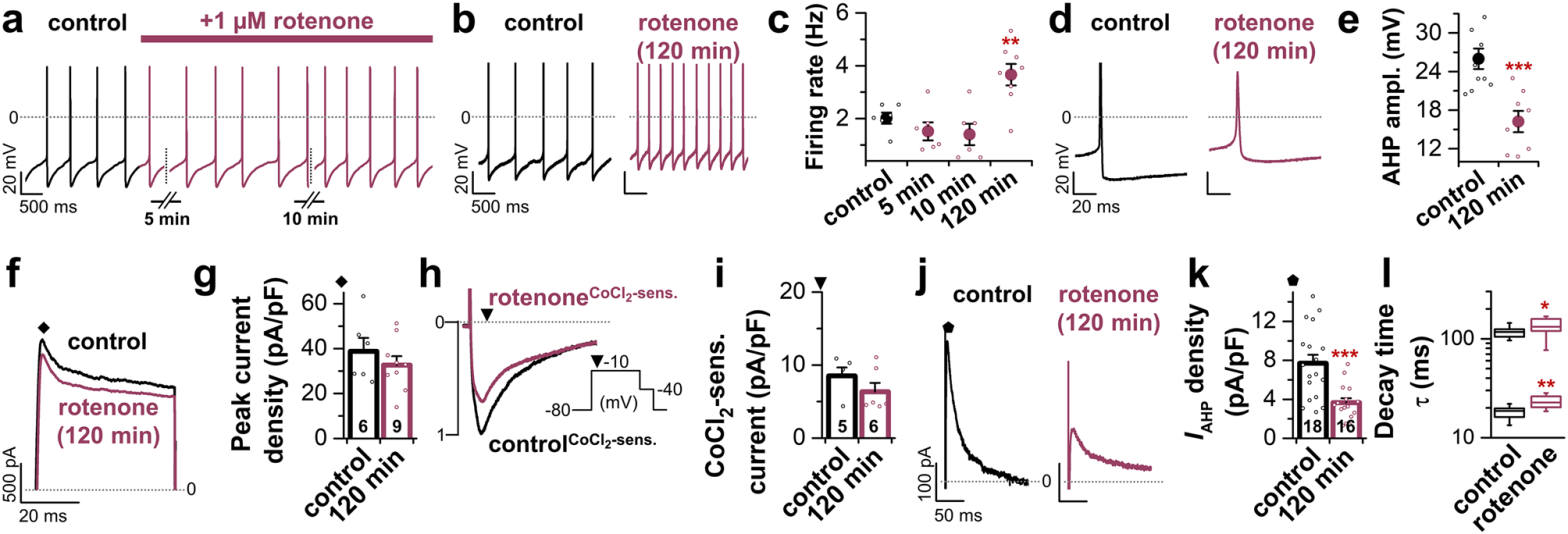
Electrophysiological alterations of LC neurons in an in vitro model of rotenone toxicity resemble those of αSyn overexpression. (**a**) Representative whole-cell spike train recording of a spontaneously active LC neuron in an acute brainstem slice derived from a C57BL/6 WT mouse. Wash-in of 1 µM rotenone after 2 min of stable firing tendentially, but not significantly, reduced the discharge rate of LC neurons within 10 min. (**b**) Example spike train recordings after slices were incubated for 2 h in standard ACSF (control, black) or ACSF + 1 µM rotenone (red) prior to recording. (**c**) Quantification of spontaneous firing 5 min (n = 6), 10 min (n = 6) and 2 h (n = 8) after exposure to 1 µM rotenone. Pre-incubation of slices in rotenone led to a significant acceleration of the spontaneous firing rate compared to control (n = 6). (**d**) Representative AP traces of a control LC neuron (black) and an LC neuron pre-incubated in rotenone (red). (**e**) Quantification revealed a significant reduction of the AHP amplitude of LC neurons in slices incubated with rotenone compared to slices incubated in control ACSF (control: n = 10, rotenone: n = 8). (**f**) Representative whole-cell voltage clamp recordings of *I*_A_ currents activated by a 500 ms voltage step to 0 mV from a holding potential of – 80 mV (as depicted by rhombus in 2a). (**g**) Peak current densities of K^+^ outward currents were ascertained from the start of the voltage step (indicated by rhombus in **f**) and did not show a significant difference between control LC neurons and rotenone-exposed LC neurons. (**h**) CoCl_2_-sensitive Ca^2+^ inward currents derived from a voltage step to − 10 mV from a holding potential of – 80 mV as depicted in the inset. Either under control conditions (black) or after 2 h incubation in 1 µM rotenone (red), 1 mM CoCl_2_ were washed in to block Cav channels. (**i**) Quantification of CoCl_2_-sensitive current densities revealed no significant difference between control and rotenone-exposed LC neurons. (**j**) Representative *I*_AHP_ currents, activated by the two-pulse voltage clamp protocol. (**k**) Quantification of *I*_AHP_ current densities revealed a drastic reduction of AHP outward currents after pre-incubation in rotenone. (**l**) In accordance with reduced medium *I*_AHP_ currents, the kinetic analysis of the decay time (τ) showed a slowing of the fast and medium decay time constants after 2 h rotenone treatment (control: n = 10, rotenone: n = 11). Box plots display median and 25/75 percentile, whiskers indicate outliers. Rest of the data are presented as mean ± SEM and also as individual data points. *p < 0.05; **p < 0.01; ***p < 0.001. [(**c**,**e**,**g**,**l**) unpaired Student’s *t*-test vs. control. (**i**) Mann–Whitney-U test vs. control. (**k**) Welch’s *t*-test vs. control].

In a first set of experiments, we performed spike train recordings in the whole-cell configuration to investigate acute effects of rotenone on the autonomous firing of LC neurons. While acute rotenone treatment for up to 10 min had no significant effect on LC firing rate (Fig. 3a,c), 120 min of preincubation with 1 µM rotenone resulted in a significant acceleration of autonomous LC firing compared to the control condition (Fig. 3b,c). Furthermore, elevated LC pacemaking was associated with alterations of AP shape (Fig. 3d) and a significant reduction of the AHP amplitude (Fig. 3e). To elucidate the underlying mechanisms, we assessed the impact of rotenone incubation on different K^+^ and Ca^2+^ currents. Peak current densities mediated by A-type K^+^ channels (Fig. 3f) were not significantly different between control neurons and rotenone-exposed LC neurons (Fig. 3g). To check whether Cav channels were affected by rotenone, we blocked Cav channels with 1 mM CoCl_2_ and compared the CoCl_2_-sensitive current of rotenone-exposed neurons to that of controls (Fig. 3h). Even though peak current densities appeared reduced after rotenone application (Fig. 3h), quantification of CoCl_2_-sensitive current densities revealed no significant difference between control and rotenone-exposed LC neurons (Fig. 3i). Strikingly, similar as for the αSyn model, we observed a significant decrease of AHP currents (Fig. 3j,k), going alongside with altered *I*_AHP_ decay time constants (τ) (Fig. 3l).

In summary, despite the fundamental differences between the αSyn and rotenone model, the electrophysiological changes induced by these two models are highly similar.

### Reduced number of SK channels available at the membrane as a cause for the rotenone-induced ***I***_AHP_ reduction

**Figure 4:**
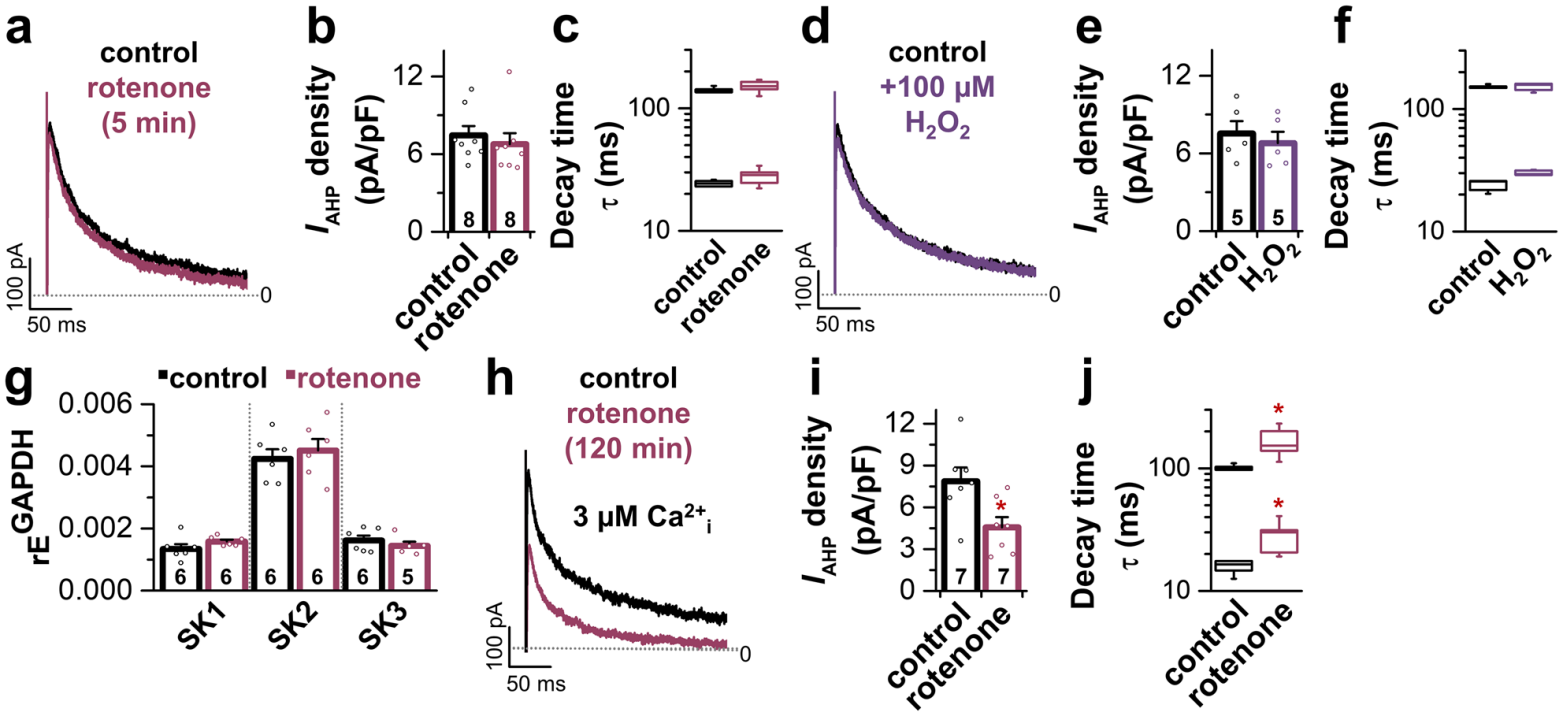
Rotenone-induced *I*_AHP_ alterations are independent of a direct rotenone effect, oxidative modulation, altered transcription and Ca^2+^ sensitivity. (**a**) Representative *I*_AHP_ recording of a LC neuron before (black) and 5 min after 1 µM rotenone was washed in (red). (**b**,**c**) Acute rotenone exposure did not alter *I*_AHP_ current density (n = 8) (**b**) or decay time (n = 6) (**c**). (**d**) Representative *I*_AHP_ recording of a LC neuron before (black) and 5 min after 100 µM H_2_O_2_ was washed in (purple). (**e**,**f**) Wash-in of 100 µM H_2_O_2_ did not alter *I*_AHP_ peak current density (**e**) or current kinetic (**f**) within 5 min of wash-in (n = 5). (**g**) Relative expression (rE) levels of SK1, SK2 and SK3 channel subunits normalized to GAPDH. For expression analysis, LC neurons were collected out of acute brainstem slices (2 h incubation in ACSF (control) or 2 h incubation in 1 µM rotenone). (**h**) Representative AHP currents in LC neurons of control slices and slices after 2 h pre-incubation with 1 µM rotenone using high concentrations (3 µM) of free Ca^2+^ in the pipette solution to achieve a full SK channel activation. (**i**,**j**) Use of high [Ca^2+^]_i_ did not restore the rotenone-induced reduction of *I*_AHP_ current density (n = 7) (**i**) or current kinetic (n = 5) (**j**). Box plots display median and 25/75 percentile, whiskers indicate outliers. Rest of the data are represented as mean ± SEM and also as individual data points. *p < 0.05. [(**b**,**c**) Mann–Whitney-U test vs. control. (**e**,**f**,**g**,**i**): unpaired Student’s *t*-test vs. control. (**j**): Welch’s *t*-test vs. control].

In a next set of experiments, we aimed at shedding light on the mechanism of how long-term exposure to rotenone affects AHP/SK currents. First, to check whether rotenone acts as a direct modulator of SK channels, we recorded AHP currents of LC neurons before and 5 min after wash-in of 1 µM rotenone. The quantification of peak current densities showed that acute rotenone exposure did not alter the *I*_AHP_ current density (Fig. 4a,b). Also, the decay of *I*_AHP_, which followed a bi-exponential time course, representing a “fast” and a “medium” component, was not changed due to the rotenone application (Fig. 4c). Rotenone is known to depolarize the mitochondrial membrane potential and to induce the release of ROS (H_2_O_2_)^30^. A study using adrenal medullary chromaffin cells demonstrated that SK2 and SK3 channels are modulated by the associated cellular redox state^31^. Given that murine LC neurons robustly express both SK2 and SK3 channels^22^, we subsequently investigated whether rotenone exerts an indirect effect on these channels by changing the cellular redox state. However, wash-in of 100 µM H_2_O_2_ did not alter the *I*_AHP_ peak current density or τ within 5 min of wash-in (Fig. 4d–f). Next, we elucidated whether long-term rotenone incubation of slices changed the expression level of the SK channel subunits SK1, SK2 and SK3. Hence, we performed qPCR analysis with RNA that was obtained from LC neurons, which were pipette-picked from slices that were incubated for 120 min either in ACSF (control) or in 1 µM rotenone. The relative expression levels of SK1, SK2, and SK3 remained unchanged after 120 min rotenone exposure (Fig. 4g). As SK channels are regulated by changes of the intracellular Ca^2+^ ([Ca^2+^]_i_) concentration, we tested whether the rotenone-induced *I*_AHP_ reduction and changes in the decay time constants can be revoked by a high concentration of free Ca^2+^ (3 µM) in the pipette solution, a concentration high enough to fully activate all SK channels available at the plasma membrane. However, the use of high [Ca^2+^]_i_ did neither restore the rotenone-induced reduction of *I*_AHP_ current density (Fig. 4h,i versus Fig. 3j,k) nor the decelerated τ (Fig. 4j versus Fig. 3l). Thus, as high [Ca^2+^]_i_ could not rescue the reduced *I*_AHP_ current amplitudes, we postulate that long-term rotenone treatment does not alter the gating or Ca^2+^-sensitivity of the channels and thus rotenone must act by reducing the quantity of SK channels on the plasma membrane of LC neurons. A reduced number of SK channels at the plasma membrane could explain the reduction of the *I*_AHP_ peak current density, changes to the kinetics of the *I*_AHP_ and the reduced AHP amplitude of action potentials of LC neurons after long-term rotenone treatment.

### SK channel activation with NS309 ameliorates rotenone-induced alterations of LC neurons

In a recent study, we have shown that pharmacological activation of SK channels by NS309^32^ decreases LC pacemaking^22^. In addition, activation of SK channels with NS309 is associated with a reduction of the loss of dopaminergic neurons after rotenone^33,34^ or glutamate treatment^35,36^. Therefore, we hypothesized that activation of SK channels by NS309 treatment would counteract the increased pacemaking caused by rotenone, by restoring macroscopic SK channel conductivity of LC neurons.

**Figure 5:**
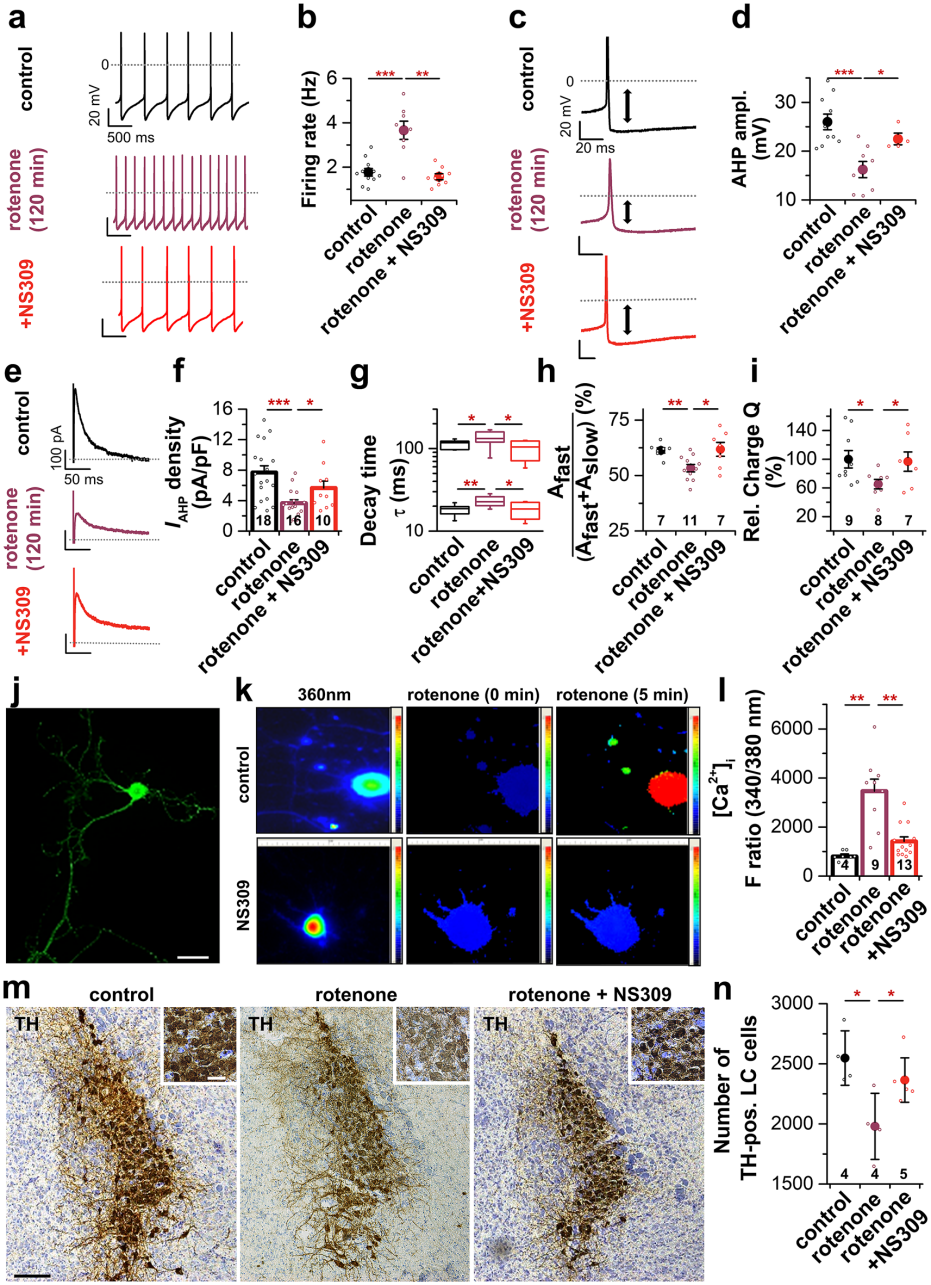
SK channel activation with NS309 prevents the rotenone-induced increase of firing frequency, rotenone-induced Ca^2+^ overload in LC neurons and loss of TH-immunoreactive LC neurons. (**a**) Representative spike trains of LC neurons under control conditions (black) and after 2 h pre-incubation with 1 µM rotenone (red) or 1 µM rotenone and 20 µM NS309 (bright red). (**b**) Quantification of AP firing frequency revealed that rotenone incubation (n = 8) led to a two-fold increase of the firing rate compared to control (n = 11), which was prevented by simultaneous activation of SK channels with NS309 (n = 9). (**c**) Representative AP of an LC neuron after simultaneous incubation with rotenone and NS309. (**d**) Analysis of the AHP amplitude revealed that NS309 significantly diminished the rotenone-induced reduction of the AHP (control: n = 10, rotenone: n = 8, rotenone + NS309: n = 4). (**e**) Representative voltage clamp recording of *I*_AHP_ currents after simultaneous incubation of the slice with rotenone and NS309. (**f**) The rotenone-induced reduction of *I*_AHP_ current density was prevented by simultaneous application of NS309. (**g**,**h**) *I*_AHP_ current kinetic analysis showed that the co-application of NS309 almost fully restored the rotenone-induced increase in decay time (control: n = 10, rotenone: n = 10, rotenone + NS309: n = 7). (**i**) Quantification of the net charge flux Q revealed that by simultaneous activation of SK channels with NS309 the rotenone-induced decrease in charge efflux was prevented. (**j**) Illustration of a primary LC neuron cultured for 1 week and stained with an anti-TH primary and Alexa488-conjugated secondary antibody. Scale bar: 100 µm. (**k**) Representative Ca^2+^ imaging of control cells (upper row) and cells pre-incubated with 20 µM NS309 (lower row). 5 min after application of 1 µM rotenone, a drastic increase of [Ca^2+^]_i_ (indicated by color change from blue to red) was detected in control cells, whereas NS309 pre-treated cells did not show any alteration of fluorescence intensities after rotenone application. (**l**) Quantification of intracellular Ca^2+^ by the ratio of fluorescence intensities of Ca^2+^-bound fura-2 (excitation at 340 nm wavelength) and free fura-2 (excitation at 380 nm wavelength) before application of rotenone (control), 5 min after application of rotenone and 5 min after application of rotenone following pre-incubation with 20 µM NS309. SK channel activation with NS309 protected primary LC neurons from rotenone-induced Ca^2+^ overload. (**m**) Representative images of TH-immunoreactive (TH-ir) cells of the LC from the right hemisphere. Prior to sectioning, brainstem blocks containing the LC region were incubated in ACSF (control), ACSF + 1 µM rotenone, or ACSF + 20 µM NS309 + 1 µM rotenone for 5 h. Scale bar: 100 µm, inset: 20 µm. (**n**) Unbiased stereology of TH-positive LC-neurons. SK channel activation with NS309 significantly reduced rotenone-induced neuronal loss in the LC. Box plots display median and 25/75 percentile, whiskers indicate outliers. Rest of the data are presented as mean ± SEM and also as individual data points. *p < 0.05; **p < 0.01; ***p < 0.001. [(**b**) rotenone vs. control, unpaired Student’s *t*-test/rotenone vs. rotenone + NS309, Welch’s *t*-test/rotenone + NS309 vs. control, unpaired Student’s *t*-test. (**d**,**h**) rotenone vs. control & rotenone + NS309, unpaired Student’s *t*-test/rotenone + NS309 vs. control, Welch’s *t*-test. (**f**,**i**) rotenone vs. control, Welch’s *t*-test/rotenone vs. rotenone + NS309, unpaired Student’s *t*-test/rotenone + NS309 vs. control, unpaired Student’s *t*-test. (**g**) decay time tau_slow_: rotenone vs. control & rotenone + NS309, unpaired Student’s *t*-test/rotenone + NS309 vs. control, Welch’s *t*-test; decay time tau_fast_: Student’s *t*-test. (**h**) rotenone vs. control & rotenone + NS309, unpaired Student’s *t*-test/rotenone + NS309 vs. control, Welch’s *t*-test. (**l**) rotenone vs. control, unpaired Student’s *t*-test/rotenone vs. rotenone + NS309, Mann–Whitney-U test/rotenone + NS309 vs. control, Mann–Whitney-U test. (**n**) rotenone vs. control & rotenone + NS309, unpaired Student’s *t*-test].

In a first set of experiments, we performed patch clamp recordings of LC neurons from slices that were either incubated for 2 h in ACSF (control), 1 µM rotenone or 1 µM rotenone + 20 µM NS309 (Fig. 5a). Consistent with the results described above, we observed a two-fold increase of the firing rate due to the incubation with rotenone. Notably, simultaneous activation of SK channels with NS309 prevented the rotenone-induced acceleration of firing (Fig. 5b) and significantly diminished the rotenone-induced reduction of the AHP (Fig. 5c,d). In addition, analysis of *I*_AHP_ peak current densities showed that the marked rotenone-induced reduction of *I*_AHP_ was prevented by simultaneous administration of NS309 (Fig. 5e,f). In accordance with these results, preincubation with NS309 had a preventive effect on the decay time constants of *I*_AHP_ and the relative *I*_AHP_ charge, which resulted in values comparable to control (Fig. 5g,i). Quantification of the amplitude ratio of the decay time constants revealed a reduced “fast” component of *I*_AHP_ due to rotenone incubation, which was also restored to control values by co-incubation with NS309 (Fig. 5h).

Next, we used primary LC neuronal cultures to investigate whether acute rotenone exposure alters the [Ca^2+^]_i_ level of LC neurons. It is known that the neurotoxic effect of rotenone is partly due to a dysregulated Ca^2+^ homeostasis and resulting oxidative stress^37^. Therefore, calcium imaging experiments in primary LC neurons were performed using the Ca^2+^ marker Fura-2 AM (Fig. 5j–l). To determine the [Ca^2+^]_i_, neurons were excited with wavelengths of 340 nm (reflecting Ca^2+^-bound fura-2) and 380 nm (reflecting free fura-2) and the fluorescence intensities were recorded. The ratio of these intensities (F ratio) served as a measure of the [Ca^2+^]_i_ concentration. The representative images and quantification of the F ratio show that 5 min after application of 1 µM rotenone, a drastic increase of [Ca^2+^]_i_ was detected in control cells, whereas cells pre-treated with 20 µM NS309 did not show any alteration of fluorescence intensities after rotenone application (Fig. 5k,l). Hence, SK channel activation by NS309 protected primary LC neurons from rotenone-induced Ca^2+^ overload. Note that we have also performed control experiments, which show that the observed Ca^2+^ increase was not mediated by enhanced influx via plasmalemmal Ca^2+^ channels (Supplementary Fig. S4).

Moreover, we further investigated whether the activation of SK channels is not only beneficial for preventing electrophysiological changes of LC neurons, but also providing a neuroprotective effect. Therefore, the impact of SK channel activation on the rotenone-induced LC cell loss was evaluated with unbiased stereological quantification of TH-immunoreactive (TH-ir) LC neurons. The TH-ir LC cells were identified with DAB staining and Nissl counterstaining (Fig. 5m). As expected a significant degeneration of TH-ir LC cells was measured after 5 h incubation with rotenone (Fig. 5m,n). Strikingly, NS309 significantly diminished the rotenone-induced neuronal cell loss in the LC when applied simultaneously (Fig. 5n). These data show that the reduced SK channel conductivity at the plasma membrane cause increased action potential firing patterns which are associated with the loss of neurons in the LC. SK channel activators might therefore be beneficial to protect LC neurons at the early stages of PD.

## Discussion

Dysfunction and neurodegeneration of noradrenergic LC neurons is a common feature of PD and other neurodegenerative diseases. Despite the early involvement in the prodromal phase of PD, only little is known about the functional consequences LC neurons face during the course of PD. Within this study, we aimed to elucidate the electrophysiological characteristics of LC neurons when rendered parkinsonian by either focal overexpression of human αSyn^WT^ or mutated αSyn^A53T^, or by application of rotenone, a complex I inhibitor known to induce PD pathology in mice^38^. Interestingly, despite the different nature of these two PD models, we observed very similar electrophysiological alterations and channel dysfunctions in both models. Three main conclusions can be drawn from our results. First, although SK channels do not control LC neuron pacemaking alone, their functionality is essential for slow and regular LC firing. Second, overexpression of αSyn as well as rotenone treatment disturb SK channel function and lead to an elevated AP firing frequency of LC neurons, which is associated with severe alterations of the AHP amplitude. Third, SK channels might present pharmacological targets for neuroprotective therapies given the fact that the SK channel activator NS309 showed neuroprotective effects in the rotenone model by decreasing the spontaneous LC firing rate to a physiological level and preventing subsequent neuronal loss in the LC following in vitro rotenone exposure. However, we did not provide an in vivo proof that SK channel activators are ultimately helpful against the PD progression. Future independent in vivo studies, addressing whether SK activators like NS309 can prevent Lewy body-like pathology in LC neurons, would strengthen our current hypothesis.

Given the fact that LC neurons have features which are very similar to that of the highly vulnerable dopaminergic SN neurons, including long and highly branched axons, a large number of neurotransmitter release sites, slow continuous autonomous pacemaking, and notable dendritic Ca^2+^ oscillations^3,20^, one can imagine that LC neurons also have high bioenergetic requirements to sustain their electrophysiological and functional activity. Moreover, previous work has shown that steady LC pacemaking triggers Ca^2+^-induced mitochondrial oxidant stress, which was significantly elevated during enhanced LC firing under hypercapnia^20^. Our results show that αSyn overexpression and long-term rotenone exposure also lead to elevated LC pacemaking, likely triggering similar effects. Based on the early formation of Lewy pathology in LC neurons, starting during the prodromal stage of PD, αSyn-induced hyperexcitability likely increases the bioenergetic burden of LC cells and could thereby be a major driver of subsequent LC neurodegeneration.

During LC pacemaking, Ca^2+^ influx—likely via L- and T-type Ca^2+^ channels—activates SK channels which mediate K^+^ outward currents that flow during the AHP (*I*_AHP_) and thus SK channels, primarily SK2, control the medium AHP (mAHP) current^22^, the main current component that we recorded in our *I*_AHP_ mesurements. The *I*_AHP_ is critical for regulating autonomous pacemaking, as inhibition of SK channels leads to a decreased mAHP and increased neuronal excitability^39^. Our results are consistent with these previous observations and extend them in several aspects. αSyn overexpression, as well as long-term rotenone exposure, led to the same dysfunction of these channels: an increased AP frequency induced by a decreased post-hyperpolarization current (*I*_AHP_) after long-term exposure. The most straightforward explanation of our results is that the reduced *I*_AHP_ results from a reduced or disturbed membrane trafficking of the SK channels. Although the precise mechanisms linking αSyn overexpression and rotenone exposure to SK channel dysfunction remain to be investigated in detail, support for this hypothesis comes from the fact that the transcripts levels of the SK channel subtypes SK1, SK2 and SK3 was not altered by rotenone. Most importantly, activation of the remaining SK channels with the SK channel activator NS309 (20 µM) or high concentrations of intracellular Ca^2+^ (3 µM) were not able to restore the *I*_AHP_ current amplitudes, indicating that it is not an altered gating of the Ca^2+^ gated channels and that it must be a reduced number of Ca^2+^-dependent channels at the plasma. The medium *I*_AHP_ and the pacemaking of LC neurons critically depend on SK2 channels^22^. We found reduced *I*_AHP_ currents in LC neurons of two different PD models, which is in perfect agreement with studies reporting (1) that genetic deletion in the SK2 gene reduces *I*_AHP_, resulting in a PD mouse model^40^ and (2) that heterozygous loss-of-function mutations (haploinsufficiency) in *KCNN2*, encoding SK2, lead to novel autosomal dominant neurodevelopmental movement disorders^41^.

While the results of our rotenone exposure experiments showed a clear increase of firing frequency, mediated by SK channel dysfunction, Yee and colleagues^42^ observed a rotenone-induced, dose-dependent decrease of autonomous LC pacemaking. Importantly, in contrast to our long-term rotenone application over 120 min, these experiments investigated acute effects of rotenone (30 min wash-in)^37,42^. The different time course could very well explain the differences between these two models. In fact, also in our experiments we observed a tendency for a firing frequency decrease after 5 and 10 min rotenone wash-in. We hypothesize that acute exposure of rotenone to LC neurons may result in intracellular Ca^2+^ overload, which could open K_ATP_ channels and thereby decrease LC pacemaking. Another alternative explanation that needs to be considered is the bioenergetic effect of acute blockade of the electron transport chain (ETC) by rotenone-induced complex I inhibition. ETC inhibition leads to elevated levels of ADP which also trigger opening of K_ATP_ channels and a decrease of firing frequency^43^.

PD pathology within the noradrenergic LC system is tightly linked to several non-motor symptoms in the prodromal phase of PD patients^44,45^. However, it has been difficult to ascertain if these symptoms stem from early dysfunction of Lewy pathology harboring LC cells or from subsequent neurodegeneration and NE deficiency. Our observations, suggesting LC hyperactivity prior to neurodegeneration, seem to be of particular interest regarding at least two prominent and early non-motor symptoms, anxiety and depression. There is good evidence from previous studies which demonstrates that increased LC activity is anxiogenic^46^ and can cause depressive behavior in rodents^4^. Despite the need for further investigation, our results suggest that Lewy pathology formation in LC neurons could mediate anxiety and depression in prodromal PD patients. Given the clinical relevance of LC hyperactivity on the level of symptomatology and the probably negative pathophysiological consequences on the cellular side, SK channel activators may present new therapeutic targets to ameliorate LC hyperactivity in prodromal PD. The frequency and thus the oxidative stress in LC neurons could be arbitrarily titrated by SK2 activators and blockers independent of the pathogenesis (toxic or genetic) of the disease. Our data show that SK channel activation via NS309 protected primary LC neurons from rotenone-induced Ca^2+^ overload and significantly counteracted degeneration of LC cells in in situ brain tissue. However, one caveat that should be considered is the robust expression of SK channels in the dopaminergic SN. Since SK channel activators slow SN pacemaking and decrease dopamine release^47^, they could potentially worsen motor symptoms, especially in late stage PD patients. This highlights the need to further investigate if SK channel dysfunction is isolated to the noradrenergic LC, or a general feature of PD pathogenesis affecting also other vulnerable brain regions like the SN.

Due to the demographic change in the upcoming decades, the prevalence of PD will increase further if a disease modifying treatment is not discovered and implemented. Identification of new therapeutic targets, which would allow slowing, stopping or reversing the progression of PD already during the prodromal phase, presents an urgent need. By using a wide array of different electrophysiological readouts, we were able to link αSyn- and rotenone-induced LC pathology to dysfunction of SK channels, thereby providing a putative new target for therapeutic intervention in the prodromal phase of PD.

## Methods

### Animals

Wild-type, male C57BL/6 N mice were purchased from Charles River (Sulzfeld, Germany) and were 8 weeks old at the beginning of the experiments. Mice were housed in individually ventilated cages under a 12 h/12 h light–dark cycle with ad libitum access to food and water. The health status of the animals was checked regularly by animal care technicians and a veterinarian. All procedures involving mice were conducted in accordance with protocols approved by the Regierungspräsidium Giessen, Germany V54-19 c 20 15 h 01 MR 20/15 Nr. 66/2015. The investigation conforms to the principles outlined by the Declaration of Helsinki and to the guide for the Care and Use of Laboratory Animals (NIH Publication 85-23). Experiments adhered to guidelines from the Guide for the Care and Use of Laboratory Animals, U.S. Department of Health and Human Services.

### Primary neuronal cultures from LC

Wild-type C57BL/6N pups (3–5 days old) were anesthetized with isoflurane, decapitated and brains were harvested in cold HBSS (Invitrogen). Coronal, 400 µm thick brainstem sections containing the LC region were made using a Campden 7000smz2 vibratome (Campden Instruments). The microdissection followed a protocol described previously by Johnson et al*.*^48^. First, the LC region was removed from the brain with sterile razor blades. The microdissection was performed in Hibernate A medium (Invitrogen, Gibco) supplemented with 1 × B27 (Invitrogen, Gibco) and 0.5 mM glutamine (Invitrogen, Gibco). Subsequently, brain tissue was digested with trypsin (Invitrogen, Gibco) at 37 °C for 30 min, followed by the inactivation with trypsin inhibitor. Cells were dissociated and plated on coated dishes with polyethylenimine. The neuronal differentiation was performed in Neurobasal A medium (Invitrogen) supplemented with 1 × B27, 0.5 mM glutamine, 50 ng/ml BDNF (PeproTech) and 30 ng/ml GDNF (Sigma). Cultures were left in the incubator at 37 °C at 5% CO_2_ for at least 7 days to mature with a media change twice a week (one quarter of the medium) with fresh Neurobasal A, 1 × B27 and 0.5 mM glutamine without BDNF and GDNF.

### rAAV vectors and stereotactic injection

Recombinant adeno-associated viral (rAAV) vectors, serotype 1/2, were used to overexpress either human wild-type (WT)-αSyn (rAAV1/2-CMV/CBA-human-WT-αSyn-WPRE-BGH-pA (rAAV1/2- αSyn^WT^); viral titer 5.1 × 10^12^ gp/ml, purchased from GeneDetect) or human mutant-A53T-αSyn (rAAV1/2-CMV/CBA-human-A53T-αSyn-WPRE-BGH-pA (rAAV1/2-αSyn^A53T^); viral titer 5.1 × 10^12^ gp/ml, purchased from GeneDetect) and—as control—luciferase (rAAV1/2-CMV/CBA-luciferase-WPRE-BGH-pA (rAAV1/2-luc), viral titer 5.0 × 10^12^ gp/ml, purchased from GeneDetect). Expression of target proteins was driven by a chicken beta actin (CBA) promoter combined with a cytomegalovirus (CMV) immediate early enhancer sequence. To assess a high transcription rate, the woodchuck post-transcriptional regulatory element (WPRE) was included into the vectors^49,50^. For stereotactic injection of the rAAV vectors into the right LC region, mice were anesthetized with 100 mg/kg ketamine and 5 mg/kg xylazine. A volume of 1.25 µl of each vector was injected with a velocity of 125 nl/min using a microinjector (UltraMicro Pump UMP3, World Precision Instruments) based on the following coordinates: medio-lateral − 0.9 mm, antero-posterior − 5.4 mm and dorso-ventral − 3.65 mm relative to Bregma^51^.

### Electrophysiology

For slice preparation, mice were anesthetized with isoflurane, decapitated and brains were removed rapidly. Mice older than 30 days were injected intra-peritoneally with ketamine/xylazine (Sigma-Aldrich) anesthesia (260 and 40 mg per kg body weight) and perfused transcardially with an ice-cold preparation solution composed of (in mM): 2.5 KCl, 1.25 NaH_2_PO_4_, 10 MgSO_4_, 20 PIPES, 10 glucose, 200 saccharose, and 0.5 CaCl_2_, pH 7.35 with NaOH prior to decapitation. Coronal sections (180–200 μm thick) were made using a Campden 7000smz-2 vibratome (Campden Instruments) in ice-cold preparation solution. After sectioning, slices were maintained in artificial cerebrospinal fluid (ACSF) composed of (in mM): 125 NaCl, 2.5 KCl, 25 NaHCO_3_, 1.25 NaH_2_PO_4_, 2 CaCl_2_, 1 MgCl_2_ and 25 glucose, equilibrated to pH 7.4 with 95% O_2_/5% CO_2_ at 30 °C. Following a 30 min resting time, slices were kept at room temperature for up to 5 h.

For patch clamp recordings, slices were transferred to a recording chamber mounted on a Zeiss Examiner.D1 microscope (Carl Zeiss Microscopy, LLC, United States) with a 40 × /0.75 phase contrast, water immersion objective and a Zeiss AxioCam MRm camera (Carl Zeiss Microscopy, LLC, United States). During recordings, slices were continuously perfused with ACSF, unless specified otherwise. During recording, LC neurons in brainstem slices were identified based on their localization at the edge of the fourth ventricle and large somata. All recordings were performed at room temperature with patch pipettes prepared from borosilicate glass capillaries GB 150TF- 8P (Science Products, Hofheim, Germany) with tip resistances between 3 and 5 MΩ. Conventional tight seal whole-cell voltage clamp or current clamp experiments were conducted with ACSF as external solution and an internal solution containing (in mM): 135 K-Gluconate, 5 KCl, 10 HEPES, 0.1 EGTA, 2 MgCl_2_, 0.2 Li_2_GTP, 2 MgATP, 3 neurobiotin and adjusted to pH 7.35 with KOH. For measurements of Ca^2+^ currents a modified ACSF with a lower CaCl_2_ concentration of 1 mM, and an internal pipette solution containing (in mM): 111 CsCH_3_SO_3_, 12.5 CsCl, 1 MgCl_2_, 1 CaCl_2_, 10 HEPES, 1 EGTA, 2 MgATP, 0.21 Na_2_GTP, adjusted to pH of 7.35 with CsOH were used. Neurobiotin (NB, Vector Laboratories) was used to label recorded neurons. To isolate autonomous spiking, all recordings were performed in the presence of synaptic blockers (1 µM CGP, 10 µM gabazine, 10 µM AP-5, 5 µM NBQX).

All patch clamp recordings were made using an Axopatch 200B amplifier (Molecular Devices, Sunnyvale CA, USA) and Clampex 10.0 software (pClamp10, Molecular Devices, Sunnyvale CA, USA). Data were digitized at 10 kHz with a Digidata 1440A digitizer (Molecular Devices, Sunnyvale CA, USA), filtered at 1–5 kHz and electrode capacitance was compensated. Membrane and tip resistances were checked after each measurement and recordings with significant changes of these values (i.e. membrane resistances < 1 GΩ and/or tip resistances of > 20 MΩ) were excluded from further analyses. For application of drugs respective stock solutions were diluted in ACSF/ modified ASCF freshly on the day of recording. As a prerequisite for application of drugs, a stable recording of at least 2 min was defined. Experiments were repeated after at least 10 ml of drug containing ACSF solution had run through the perfusion system (~ 2 min). Data were analyzed with ClampFit10 (Molecular Devices, Sunnyvale CA, USA). Drugs were stored as DMSO stocks and final DMSO concentration did not exceed 0.1%.

### Immunofluorescence staining

For immunofluorescent co-staining of NB, alpha-synuclein (αSyn) and tyrosine hydroxylase (TH) at the end of each patch clamp recording slices were transferred to a fixation solution composed of 4% paraformaldehyde (PFA) in 0.1 M phosphate buffer (PB). After fixation overnight, slices were stored in storing solution containing 30% sucrose in 0.1 M PBS. Before staining 200 µm thick slices were cut thinner (40 μm) using a cryostat microtome (Leica CM3050 S, Nussloch, Germany). Sections were washed in 0.1 M PB and blocked in 10% normal donkey serum with 0.3% Triton X-100 in 0.1 M PB for 1 h followed by overnight incubation with primary antibodies for TH (1:1000, Merck Millipore, Cat# AB152, RRID: AB_390204) and αSyn (1:1000, ThermoFisher Scientific, Cat# AHB0261, RRID: AB_2536241) at 4 °C. Subsequently, sections were washed in 0.1 M PB containing 0.3% Triton X-100 and then incubated with fluorophore-conjugated, species-specific secondary antibodies Cy3 conjugated donkey anti-mouse (1:1000, Jackson ImmunoResearch, Cat# 715-165-150, RRID: AB_2340813), Alexa488 conjugated donkey anti-rabbit (1:1000, Invitrogen, Cat# A-21206, RRID: AB_2535792) and Alexa 647-conjugated streptavidin (1:1000, Jackson ImmunoResearch, Cat# 016-600-084, RRID: AB_2341101) for 2 h at room temperature in 0.1 M PB containing 0.3% Triton X-100 and 10% normal donkey serum. Before mounting, sections were washed for 25 min in 0.1 M PB containing 0.3% Triton X-100. Images were acquired using an AxioImager M2 microscope (Carl Zeiss Microscopy, LLC, United States) equipped with an ORCA-Flash4.0 LT CMOS camera (Hamamatsu C11440-42U).

### Real time qPCR

For quantitative (q-) PCR experiments, single LC neurons were pulled out of acute brainstem slices with a modified patch pipette as described previously^21^. RNA isolation was performed with the RN-easy Protect Mini Kit (Qiagen). Reverse transcription (RT) was performed with random hexamers (Roche) and Superscript II reverse transcriptase (Invitrogen) according to the instructions of the manufacturer. For further analyses, only cDNA pools that were negative for GAD, but positive for GAPDH and dopamine-beta-hydroxylase (DBH) were used.

Intron-spanning primers were designed to generate PCR products of about 110 base pairs:

- GAPDH for 5′-ACTTCAACAGCAACTCCCACTCT-3′,
- GAPDH rev 5′-GCTGTAGCCGTATTCATTGTCATA-3′;
- DBH for 5′-ACTATGTGCACTACTACCCCCAGA-3′,
- DBH rev 5′-CTCCTCACTGCTGAACCTGTTTAC-3′;
- GAD for 5′-GGAGCGGATCCTAATACTACCAA-3′,
- GAD rev 5′-GTAAGAAGCCACAGATCTTCAGG-3′;
- SK1 for 5′-GAGAAACACGTGCACAACTTCAT-3′,
- SK1 rev 5′-CAGCTCTGACACCACCTCATATG-3′;
- SK2 for 5′-GAATGACCAAGCAAATACCCTAGT-3′,
- SK2 rev 5′-GTGACGATCCTTTTCTCAAAGTCT-3′;
- SK3 for 5′- GAAAAGAGAAAGCGACTGAGTGAC-3′,
- SK3 rev 5′-CATGGAATCCTTTGAGTACAAACC-3′;

qPCRs were performed as described previously^22^. Briefly, the Platinum SYBR Green qPCR SuperMix-UDG (Invitrogen) was used and reaction mixtures were preheated at 50 °C and at 95 °C for 2 min each, followed by 40 cycles at 95 °C for 15 s, 60 °C for 30 s, and 72 °C for 30 s. Emitted fluorescence was detected online using a Mx3000P real-time PCR system (Stratagene). The amplification products for all primer pairs were confirmed by sequencing, no template control and dissociation curve analysis. Amplification efficiency was determined by analyzing the slope of a Ct/log (template concentration) plot. For normalization, primers for GAPDH were used (rE^GAPDH^ = 1/2^ΔCt^). qPCR reactions were performed in duplicate or triplicate, and control experiments in the absence of cDNA (n.c.) were included. Three independent qPCR experiments were performed.

### Calcium imaging of primary LC neurons

Primary neuronal LC cells were incubated with 2 μM FURA-2 AM for 30 min at 37 °C in HEPES-ringer buffer (HRB), containing (in mM): 136.4 NaCl, 5.6 KCl, 1 MgCl_2_, 2.2 CaCl_2_, 10 HEPES, 5 glucose and 0.1% BSA, pH 7.4. Images were acquired using a Polychrome II monochromator and an IMAGO CCD camera (Till Photonics, Martinsried, Germany) coupled to an inverted microscope (IX70; Olympus, Hamburg, Germany). Images were collected with a 20 × 0.8 numerical aperture (NA) oil immersion objective. Fluorescence intensities were monitored from single cells excited at the two wavelengths (F340 and F380) and the emission wavelength used was 510 nm. An increase in intracellular Ca^2+^ was reflected by a fluorescence increase when exciting at 340 nm and a corresponding decrease when excited at 380 nm. F340 and F380 were recorded separately and combined (fluorescence ratio: r = F340/F380) after background subtraction (fluorescence of a cell-free area).

### Stereology

To quantify TH-immunoreactive LC neurons, 30 µm thick tissue sections were stained against TH with 3,3-diaminobenzidine (DAB), counterstained with cresyl-violet and the optical fractionator workflow (StereoInvestigator version 8, MicroBrightField Biosciences) was used^18^. For DAB stainings, free-floating brainstem sections containing the LC region were washed three times in 0.1 M PB and quenched with 3% H_2_O_2_ and 10% methanol for 15 min. Then, sections were blocked with 5% normal donkey serum/0.3% Triton X-100 in 0.1 M PB for 1 h followed by overnight incubation with a primary anti-TH antibody (1:1000, Merck Millipore, Cat# AB152, RRID: AB_390204) at 4 °C in the same blocking solution. Subsequently, sections were washed in 0.1 M PB for 20 min and then incubated with a biotinylated anti-rabbit secondary antibody (1:1000, Jackson ImmunoResearch, Cat# 711-065-152, RRID: AB_2340593) for 1 h, followed by incubation in avidin–biotin-peroxidase solution (ABC Elite, Vector Laboratories) for 1 h before initiating the color reaction with 5% DAB (Serva), diluted in 0.1 M PB with 0.02% H_2_O_2_. DAB-stained sections were counterstained with cresyl-violet. Brightfield images were acquired using an AxioImager M2 microscope (Carl Zeiss Microscopy, LLC, United States) equipped with an Axiocam 506 color camera (Carl Zeiss Microscopy, LLC, United States).

To quantify LC cell numbers four systematically, randomly acquired sections per animal containing the complete rostro-caudal extent of the LC region were analyzed. First, contours including all TH-positive neurons of the LC were drawn in 5 × magnification using a Microphot-FX microscope (Nikon, Tokyo, Japan). Counting was performed in 40 × magnification using the following parameters: grid size 100 × 100 μm, counting frame 85 × 85 μm, and guard zones 2 μm.

### Data and statistical analysis

Data are reported as mean ± standard error of the mean (SEM) and were analyzed using Excel (Microsoft, Washington, USA), Clampfit (Molecular Devices, California, USA) and OriginPro (OriginLab Corp., Guangzhou, China. Data sets were tested for normality using the Shapiro Wilk test and for variance homogeneity using the one-way ANOVA—Levene’s test. In normally distributed data sets, statistical significance was calculated using paired or unpaired Student’s *t*-test, or Welch’s *t*-test depending on the variance. Otherwise, non-parametric statistical tests, e.g. the Mann–Whitney-U test or the Mood’s median test were used depending on the variance. Decay time constants (τ) were determined by fitting the *I*_AHP_ current tails to a bi-exponential decay function. Box plots display median, 25^th^ and 75^th^ percentile, whiskers indicate the total range. All experiments were performed at room temperature (21–24 °C). * indicates *p* < 0.05; ** when *p* < 0.01 and *** when *p* < 0.001. Only significant changes are indicated in the figures and a lack of asterisks next to the bar graphs shows that there are no significant changes.

### Materials

All drugs or chemicals were obtained from Sigma-Aldrich, Carl Roth, Honeywell Fluka or biomol.

### Ethical approval

All animal research was ethically approved by the governmental authority (Regierungspräsidium Giessen, Germany reference V54-19 c 20 15 h 01 MR 20/15 Nr. 66/2015). All procedures involving mice were performed in accordance with relevant guidelines and regulations. The study is reported in accordance with ARRIVE guidelines.

## Supplementary Information

Supplementary Figures.

## Data Availability

The data supporting the findings of this study are available from the corresponding author upon reasonable request.
