# Supplementary material for: Enhanced firing of locus coeruleus neurons and SK channel dysfunction are conserved in distinct models of prodromal Parkinson’s disease
Source: Sci Rep. 2022 Feb 24;12:3180. doi: 10.1038/s41598-022-06832-1 (PMC8873463; doi:10.1038/s41598-022-06832-1)
Supplement: Supplementary file 1 — Supplementary Figures. [file 41598_2022_6832_MOESM1_ESM.pdf]

Supplementary Information

Enhanced firing of locus coeruleus neurons and SK channel dysfunction are conserved in distinct models of prodromal Parkinson's disease

Lina A. Matschke[1,2,§], Marlene A. Komadowski[1,§], Annette Stöhr[1], Bolam Lee[2], Martin T. Henrich[2], Markus Griesbach[1], Susanne Rinné[1], Fanni F. Geibl[2], Wei-Hua Chiu[2], James B. Koprich[3], Jonathan M. Brotchie[3], Aytug K. Kiper[1], Amalia M. Dolga[4], Wolfgang H. Oertel[2,5,†], and Niels Decher[1,†,*]

[1]Institute for Physiology and Pathophysiology, Vegetative Physiology and Marburg Center for Mind, Brain and Behavior - MCMBB, Philipps-University Marburg, 35037 Marburg, Germany.

[2]Clinic for Neurology, Philipps-University Marburg, 35043 Marburg, Germany.

[3]Krembil Research Institute, Toronto Western Hospital, University Health Network, 8KD402, Toronto, ON, M5T 2S8, Canada.

[4]Faculty of Science and Engineering, Groningen Research Institute of Pharmacy, Department of Molecular Pharmacology, University of Groningen, 9713 AV Groningen, The Netherlands.

[5]Hertie Senior Research Professor of the Charitable Hertie Foundation, 60323 Frankfurt/Main, Germany.

[§]Matschke LA and Komadowski MA are joint first author

[†]Oertel WH and Decher N are shared senior authors

*Decher N is corresponding author

Prof. Dr. Niels Decher
Institute for Physiology and Pathophysiology
Philipps-University Marburg
Deutschhausstraße 2
35037 Marburg, Germany
T: + 49-6421-28-62148
ORCID 0000-0001-8892-1231
decher@staff.uni-marburg.de

Keywords: Parkinson's disease, synuclein, locus coeruleus, rotenone, patch clamp, SK channel

Supplementary Figure S1

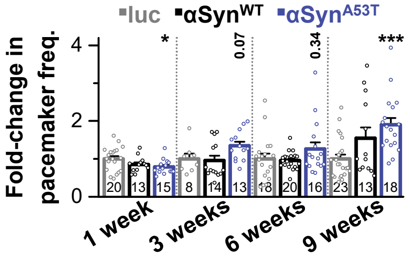

Suppl. Fig. S1. Quantification of the relative change in pacemaker frequency in spontaneously active αSyn^{WT} or αSyn^{A53T} overexpressing LC neurons at 1, 3, 6 and 9 weeks after injection. Spiking frequencies were normalized to that of the respective luc injected animals at the same time point of 1, 3, 6 or 9 weeks. Data are presented as mean ± SEM and also as individual data points. *: $p < 0.05$; ***: $p < 0.001$. *[1 week: unpaired Student's t-test vs. luc; 3 weeks: αSyn^{WT}, Mann-Whitney-U test vs. luc / αSyn^{A53T}, unpaired Student's t-test vs. luc; 6 weeks: Mann-Whitney-U test vs. luc; 9 weeks: αSyn^{WT}, Mood's median test vs. luc / αSyn^{A53T}, Mann-Whitney-U test vs. luc.]*

Supplementary Figure S2

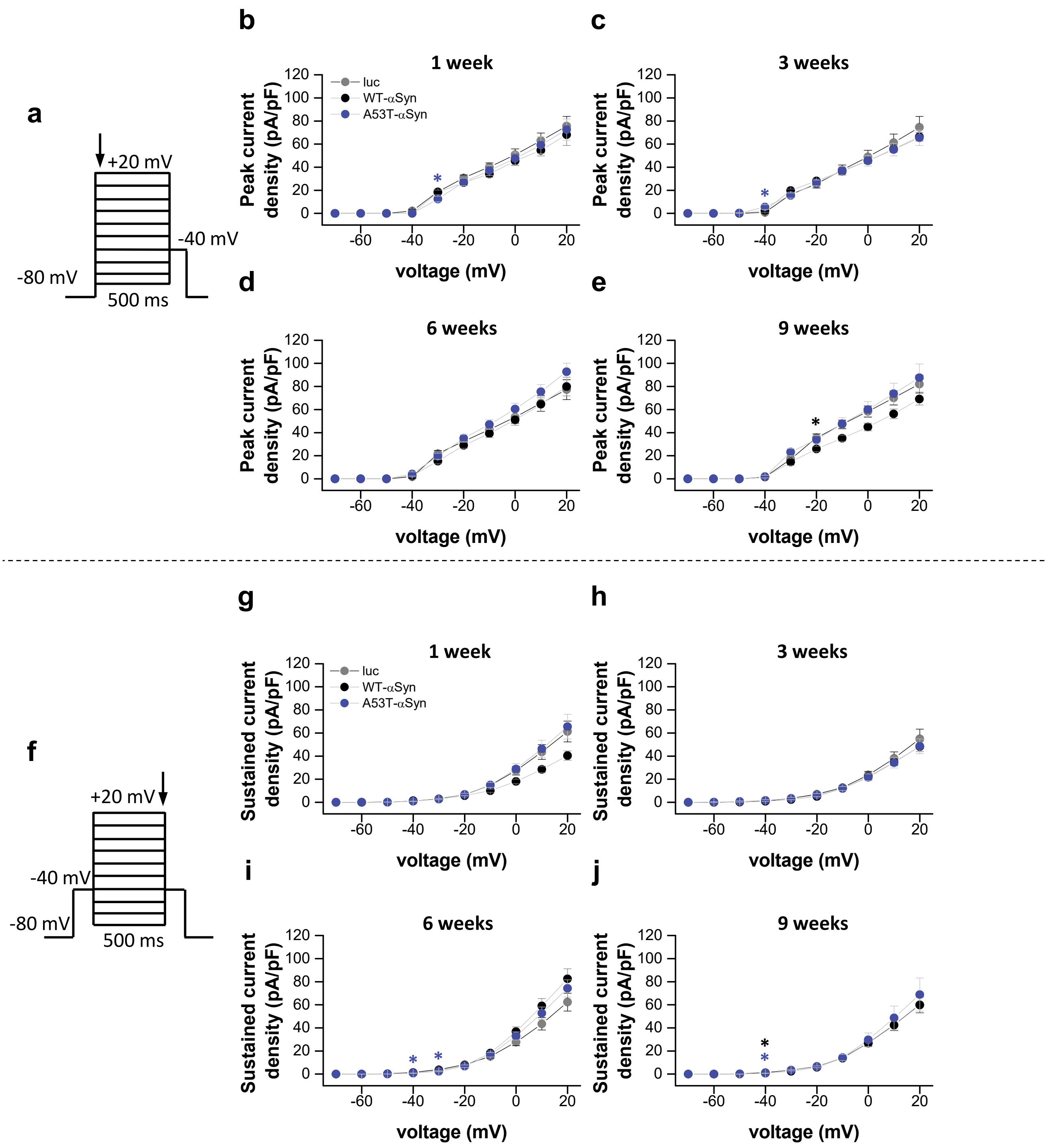

Suppl. Fig. S2. I/V Relationships of I_A and I_{sus} in LC neurons after overexpressing luc, αSyn^{WT}, or αSyn^{A53T}. (**a**) Illustration of the voltage protocol used to record rapidly inactivating K^+ outward currents (I_A). (**b–e**) I/V relationships of I_A currents after 1, 3, 6 and 9 weeks post-injection of viral vectors (luc: n = 17-20, αSyn^{WT}: n = 13-21, αSyn^{A53T}: n = 14-17). Peak currents of rapidly inactivating K^+ currents (I_A) (indicated by an arrow in **a**) were quantified for each time point after injection of viral vectors. (**f**) Scheme of the voltage protocol used to record sustained K^+ outward currents (I_{sus}). (**g–j**) I/V relationships of I_{sus} after 1, 3, 6 and 9 weeks post-injection of viral vectors (luc: n = 17-20, αSyn^{WT}: n = 12-18, αSyn^{A53T}: n = 13-15). Data of sustained K^+ outward currents (I_{sus}) were evaluated at the end of the voltage steps (indicated by an arrow in **f**) and were quantified for each time point after injection of viral vectors. Data are presented as mean ± SEM. *: $p < 0.05$. *[(b): αSyn^{A53T} at -30 mV, Mann-Whitney-U test vs. luc. (c): αSyn^{A53T} at -40 mV, Mann-Whitney-U test vs. luc. (e): αSyn^{WT} at -20 mV, Mann-Whitney-U test vs. luc. (i): αSyn^{A53T} at -40 & -30 mV, Mann-Whitney-U test vs. luc. (j): αSyn^{WT} at -40 mV, unpaired Student's t-test vs. luc / αSyn^{A53T} at -40 mV, Mann-Whitney-U test vs. luc.]*

Supplementary Figure S3

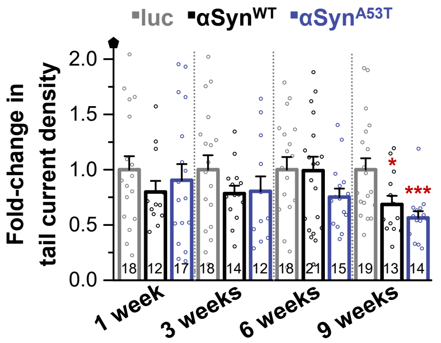

Suppl. Fig. S3. Quantification of the relative change in tail current densities in spontaneously active αSyn^{WT} or αSyn^{A53T} overexpressing LC neurons at 1, 3, 6 and 9 weeks after injection. Tail current densities were normalized to that of the respective luc injected animals at the same time point of 1, 3, 6 or 9 weeks. Data are presented as mean ± SEM and also as individual data points. *: $p < 0.05$; ***: $p < 0.001$. *[1 week: αSyn^{WT}, Mann-Whitney-U test vs. luc / αSyn^{A53T}, unpaired Student's t-test vs. luc; 3 weeks: αSyn^{WT}, Welch´s t-test vs. luc / αSyn^{A53T}, unpaired Student's t-test vs. luc; 6 weeks: αSyn^{WT}, unpaired Student's t-test vs. luc / αSyn^{A53T}, Welch´s t-test vs. luc; 9 weeks: αSyn^{WT}, unpaired Student's t-test vs. luc / αSyn^{A53T}, Mann-Whitney-U test vs. luc.]*

Supplementary Figure S4

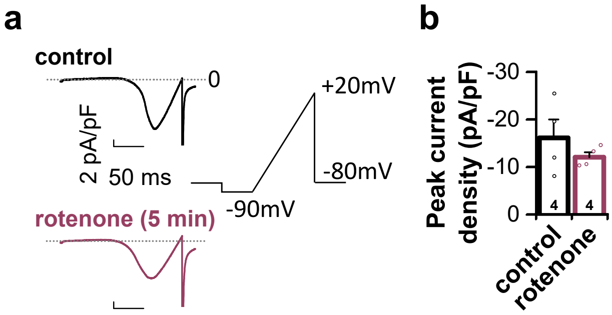

Suppl. Fig. S4. Rotenone-induced Ca^{2+} overload of LC neurons is not mediated by increased influx via plasmalemmal calcium channels. (**a**) Averaged Ca^{2+} inward currents, derived from the depicted voltage ramp protocol, under control conditions (black) and 5 min after wash-in of 1 µM rotenone (red). (**b**) Analyses of peak current densities indicate a reduction of I_{Ca} due to rotenone wash-in which was however not significant (n = 4). Data are presented as mean ± SEM and also as individual data points. *[(b) paired Student's t-test vs. control.]*
